# Tumor Treating Fields (TTFields) induce homologous recombination deficiency in ovarian cancer cells, thus mitigating drug resistance

**DOI:** 10.3389/fonc.2024.1402851

**Published:** 2024-06-27

**Authors:** Yani Berckmans, Hila M. Ene, Kerem Ben-Meir, Antonia Martinez-Conde, Roxanne Wouters, Bieke Van den Ende, Sara Van Mechelen, Roni Monin, Roni Frechtel-Gerzi, Hila Gabay, Eyal Dor-On, Adi Haber, Uri Weinberg, Ignace Vergote, Moshe Giladi, An Coosemans, Yoram Palti

**Affiliations:** ^1^ Laboratory of Tumor Immunology and Immunotherapy, Department of Oncology, Leuven Cancer Institute, KU Leuven, Leuven, Belgium; ^2^ Novocure Ltd, Haifa, Israel; ^3^ Oncoinvent AS, Oslo, Norway; ^4^ Department of Gynecology and Obstetrics, Gynecologic Oncology, Leuven Cancer Institute, KU Leuven, Leuven, Belgium

**Keywords:** Tumor Treating Fields (TTFields), ovarian cancer, drug resistance, DNA damage, synthetic lethality, conditional vulnerability, PARP inhibitors, carboplatin

## Abstract

**Background:**

Ovarian cancer is the leading cause of mortality among gynecological malignancies. Carboplatin and poly (ADP-ribose) polymerase inhibitors (PARPi) are often implemented in the treatment of ovarian cancer. Homologous recombination deficient (HRD) tumors demonstrate increased sensitivity to these treatments; however, many ovarian cancer patients are homologous recombination proficient (HRP). TTFields are non-invasive electric fields that induce an HRD-like phenotype in various cancer types. The current study aimed to investigate the impact of TTFields applied together with carboplatin or PARPi (olaparib or niraparib) in preclinical ovarian cancer models.

**Methods:**

A2780 (HRP), OVCAR3 (HRD), and A2780cis (platinum-resistant) human ovarian cancer cells were treated *in vitro* with TTFields (1 V/cm RMS, 200 kHz, 72 h), alone or with various drug concentrations. Treated cells were measured for cell count, colony formation, apoptosis, DNA damage, expression of DNA repair proteins, and cell cycle. *In vivo*, ID8-fLuc (HRP) ovarian cancer cells were inoculated intraperitoneally to C57BL/6 mice, which were then treated with either sham, TTFields (200 kHz), olaparib (50 mg/kg), or TTFields plus olaparib; over a period of four weeks. Tumor growth was analyzed using bioluminescent imaging at treatment cessation; and survival analysis was performed.

**Results:**

The nature of TTFields-drug interaction was dependent on the drug’s underlying mechanism of action and on the genetic background of the cells, with synergistic interactions between TTFields and carboplatin or PARPi seen in HRP and resistant cells. Treated cells demonstrated elevated levels of DNA damage, accompanied by G2/M arrest, and induction of an HRD-like phenotype. In the tumor-bearing mice, TTFields and olaparib co-treatment resulted in reduced tumor volume and a survival benefit relative to olaparib monotherapy and to control.

**Conclusion:**

By inducing an HRD-like phenotype, TTFields sensitize HRP and resistant ovarian cancer cells to treatment with carboplatin or PARPi, potentially mitigating a-priori and *de novo* drug resistance, a major limitation in ovarian cancer treatment.

## 1 Introduction

Ovarian cancer has the worst prognosis among gynecological malignancies. First-line standard-of-care treatment includes debulking surgery in combination with either adjuvant or neo-adjuvant treatment with a platinum-taxane doublet, mainly carboplatin and paclitaxel. This can be supplemented with therapy consisting of an angiogenesis inhibitor (bevacizumab) and/or maintenance with a poly (ADP-ribose) polymerase inhibitor (PARPi), olaparib, niraparib or rucaparib (1–3). Despite promising initial responses to therapy, approximately 80% of women experience disease progression or recurrence (1–3).

Over the last two decades, it has become well established that germline mutations and epigenetic silencing of tumor suppressor genes can be associated with a significantly elevated risk of ovarian cancer development and a more aggressive disease. Inherited mutations in BRCA related genes impair the ability of cells to repair DNA double strand breaks (DSB) through homologous recombination (HR) and their ability to support replication fork stabilization, overall leading to replication stress and genomic instability. These mutations hence create a fertile ground for the accumulation of genetic alterations and an increased likelihood of uncontrolled cell proliferation, driving cancer development. Such genes include *BRCA1*, *BRCA2*, and other genes involved in the HR pathway, traits collectively referred to as “BRCAness” (4, 5). Tumor cells possessing mutations in the HR pathway are also referred to as HR deficient (HRD) cells, as opposed to HR proficient (HRP) cells that exhibit normal expression patterns.

While individuals with HRD tumors are at elevated risk of malignant transformation, they also exhibit increased sensitivity to ovarian cancer therapies targeting DNA damage and repair mechanisms, such as platinum-based chemotherapy and PARPi, respectively (6–8). Platinum-based chemotherapy forms DNA inter- and intrastrand crosslinks, leading to stalled replication forks and consequent development of DSB. HRD cells present with conditional vulnerability to such chemotherapy drugs due to their reduced damage repair capacity leading to accumulation of DNA damage, which can induce cell death.

The particular efficacy of PARPi in HRD patients is due to the concept known as synthetic lethality, in which the individual loss of either one of two genes involved in DNA damage repair can be viable, while their simultaneous loss of activity is fatal. Inhibition of PARP impairs base excision repair (BER) activity, limiting the cell’s ability to repair DNA single strand breaks (SSB), which when left unrepaired may develop into DSB (1, 4). PARPi have further been suggested to trap the PARP enzyme within the DNA, resulting in replication fork collapse and consequent DSB formation. Accordingly, when HRD patients are treated with PARPi, synthetic lethality occurs due to deficiencies in both HR and BER pathways, the former related to the genetic predisposition of the cells, and the latter stemming from targeted inhibition by treatment with PARPi (3).

Ovarian cancer patients often present with therapy resistance after prior treatment. Several mechanisms have been suggested to explain the acquired tumor resistance to platinum-based and PARPi therapies, including dysregulation of drug influx and efflux (2, 3). Of note, acquired resistance has also been suggested to involve restoration of HR function in HRD tumors, either through secondary mutations (somatic insertion/deletion that cause a frameshift that reinstates the open reading frame) or epigenetic modifications (loss of promoter hypermethylation) (1, 3, 9). Because three out of four ovarian cancer patients are HRP (5, 10), and since patients that were initially HRD may acquire treatment resistance via transformation to an HRP-like phenotype, therapies that impose BRCAness may facilitate synthetic lethality, potentially augmenting the efficacy of PARPi.

Recently, it was shown that a state of BRCAness can be induced by Tumor Treating Fields (TTFields), a clinically approved antimitotic cancer treatment, in which electric fields are continuously and non-invasively applied to the tumor bed (11–13). Specifically, TTFields-induced downregulation of DNA repair proteins from the Fanconi Anemia (FA)-BRCA pathway has been preclinically demonstrated in several tumor types (14–17); and exploitation of this induced state of BRCAness to enhance the effects of olaparib has been shown in non-small cell lung carcinoma (NSCLC) models (14, 15). In accordance with the involvement of the FA-BRCA pathway in the repair of DNA damage induced by platinum agents (18, 19), TTFields have also been observed to augment the effect of cisplatin in preclinical models of pleural mesothelioma and NSCLC (14, 17).

TTFields therapy is currently approved in the US, Canada, China, Hong Kong, Japan, Europe, Israel, and Australia for treatment of newly diagnosed glioblastoma (GBM) concomitant with the DNA alkylating agent temozolomide; and in the US, Israel, and Europe for treatment of pleural mesothelioma concomitant with the DNA damaging agents cisplatin and pemetrexed (20–23).

The research described herein examined the efficacy of TTFields co-treatment with ovarian cancer standard therapies (24), namely carboplatin, olaparib, and niraparib, that induce DNA damage or interfere with DNA damage repair, in order to sensitize the cells to treatment owing to the plausible HRD-like state induced by TTFields. The co-treatment demonstrating highest benefit *in vitro* was also tested in an ovarian cancer animal model.

## 2 Materials and methods

### 2.1 Cell culture

The human ovarian endometrioid adenocarcinoma cell lines A2780 and A2780cis were obtained from the European Collection of Cell Cultures and from AddexBio, respectively. The human ovarian high grade ovarian serous adenocarcinoma cell line OVCAR-3 was obtained from the American Type Culture Collection (ATCC). Human cell lines were grown in RPMI media supplemented with 10% (v/v) fetal bovine serum (FBS), 1 mM sodium pyruvate, 12 mM HEPES and penicillin/streptomycin (50 µg/ml) in a 37°C humidified incubator supplied with 5% CO_2_. The media of A2780cis cells was additionally supplemented with 1 µM cisplatin (Sigma, C2210000) in every passage to maintain platinum resistance. Media and supplements were purchased from Sartorius Israel Ltd. (Biological Industries Ltd., Beit HaEmek). Murine ID8 cells were previously transduced by the Laboratory of Molecular Virology and Gene Therapy in the Leuven Viral Vector Core of KU Leuven, using a lentiviral vector (pCHMWS_CMV-fluc-I-PuroR) to create the stable luciferase producing cell line, ID8-fLuc (25). These ID8-fluc cells were cultured at 37°C with 5% CO_2_ in Dulbecco’s Modified Eagle Medium (DMEM) supplemented with 10% FCS, 100 U/ml penicillin/streptomycin, 2mM glutamine, 2.5µg/ml amphotericin B and 10 mg/ml gemcitabine, which were obtained from Gibco.

### 2.2 Application of TTFields to cells

Cells were seeded on coverslip (22 mm diameter; 20×10^3^ cells/coverslip for A2780 and A2780cis; 40×10^3^ cells/coverslip for OVCAR-3). After overnight incubation, the coverslips were transferred into inovitro^TM^ dishes containing 2 ml of media. TTFields at a frequency of 200 kHz (and intensity of 1 V/cm RMS) were applied to the cells for 72 hr using the inovitro™ system (Novocure, Haifa, Israel), as previously described (26).

### 2.3 Co-application of TTFields with drugs to cell lines

For efficacy outcomes (cell count, overall effect, and apoptosis), various concentrations of carboplatin (MCE MedChemExpress, HY-17393), olaparib (Cayman Chemical, 10621), or niraparib (Cayman Chemical, 20842) were applied, with or without TTFields.

For DNA damage and cell cycle examination, the following drug concentrations were selected: For A2780 – 6 µM carboplatin, 1 µM olaparib, and 0.5 µM niraparib; For OVCAR-3 – 16 µM carboplatin, 0.5 µM olaparib, and 0.8 µM niraparib; For A2780cis – 36 µM carboplatin, 10 µM olaparib, and 1.5 µM niraparib.

### 2.4 Cell count

Cell count was examined following treatment using Cytek Northern Lights flow cytometer (Cytek Biosciences, USA). Results are presented as percentage relative to control.

### 2.5 Overall effect

Treated cells were harvested, re-plated in 6-well plates (500 cells/well for A2780 and A2780cis; 1000 cells/well for OVCAR-3), and grown for 7 (A2780 and A2780cis) or 21 (OVCAR-3) days. Colonies were stained with 0.5% crystal violet, quantified with ImageJ, and expressed as percentages relative to control. Overall effect was calculated by multiplying colony formation with the corresponding cell count.

### 2.6 Determining the type and magnitude of TTFields-drug interactions

The surviving fraction predicted for an additive effect between TTFields and drug was calculated (per the various drug concentrations) by multiplying the actual measured surviving fractions for the individual treatments one by the other (SF_calculated additive_ = SF_TTFields_ × SF_drug_; where SF are expressed as probability) (27–29). Based on the calculated values, a trendline was determined. Additivity, synergy, or antagonism was defined when the calculated additive trendline overlapped, was above, or was below the actual measured line for TTFields+drug, respectively.

For quantifying the magnitude of TTFields-drug interaction, interaction index (I_i_) values were calculated by the Bliss independence method using mortality values (M_x_ = 1 – SF_x_) (27–29). Per the various drug concentrations, mortality predicted for an additive effect between TTFields and drug (M_calculated additive_ = M_TTFields_ + M_drug_ – M_TTFields_ × M_drug_) was divided by the actual measured mortality for TTFields+drug. Additivity was determined when the 95% confidence interval (CI) overlapped 1, synergy when 95% CI < 1, and antagonism when 95% CI > 1. Lower I_i_ values were considered indicative of higher synergy levels.

### 2.7 Apoptosis

Treated cells were stained with FITC-conjugated Annexin V (AnnV) and 7-Aminoactinomycin D (7-AAD) using a commercial kit (BioLegend, San Diego, CA, USA), according to the manufacturer’s instruction. Data acquisition and analysis were done on the Cytek Northern Lights flow cytometer.

### 2.8 Western blot analysis

Extracts were prepared from treated cells and subjected to western blot analysis (25 μg protein/sample) as previously described. Primary antibodies are outlined in **Table 1**. Horseradish peroxidase (HRP)-conjugated secondary antibody (Abcam, Cambridge, UK; cat #ab97023 or #ab6721, 1:10,000) and a chemiluminescent substrate (Immobilon Forte, Millipore, Burlington, MA, USA) were used for visualization. Bands were recorded on GeneGnome XRQ gel imager (AlphMetrix Bitech, Rödermark, Germany). Densitometric readings were normalized to GAPDH with FIJI software and expressed as fold change relative to control.

**Table 1:** Primary antibodies used in the study for western blot analysis.

Antigen	Vendor	Catalog #	Dilution
BRCA2	Cell Signaling	10741	1:1000
FANCB	Cell Signaling	14243	1:1000
FANCD2	Cell Signaling	16323	1:1000
FANCJ	Cell Signaling	4578	1:1000
p21	Santa Cruz	SC-6246	1:500
GAPDH	Santa Cruz	SC-32233	1:1000

### 2.9 DNA damage examination

Treated cells were fixed with 4% paraformaldehyde for 10 min, permeabilized for 20 min with 0.5% Triton X-100 in PBS, and blocked with donkey serum (PBS with 0.3% Triton X-100 and donkey serum 1:100). Cells were incubated at 4°C overnight with anti-ɣH2AX antibody (Cell Signaling, Danvers, MA, USA; #9718, 1:400), followed by incubation at room temperature for 1 hr with Alexa Flour 488-conjugated secondary antibody (Jackson Immunoresearch, Cambridge, UK; #711–545-152, 1:500) and 0.2 μg/ml 4’,6-diamidino-2-phenylindole (DAPI; Sigma Aldrich, Rehovot, Israel). LSM 700 laser scanning confocal system (Zeiss, Gottingen, Germany) was utilized to obtain images, and the mean number of foci per nucleus was determined using the FIJI software with the BioVoxxel plugin.

### 2.10 Cell cycle analysis

Treated cells were fixed with 70% ice-cold ethanol for 30 min, pelleted, washed, and stained for 30 min at 37°C in phosphate buffered saline (PBS) containing 1% FBS 5 µg/ml 7-AAD (BioLegend), 200 µg/ml RNase, 1 mM EDTA and 0.1% Triton X-100. Data acquisition (at 665/30 nm) and analysis were done on the Cytek Northern Lights flow cytometer and the FlowJo 10.8.1 software (BD Biosciences), respectively.

### 2.11 Co-application of TTFields and olaparib *in vivo*

Murine experiments were approved by the KU Leuven ethical committee (P082/2021). NIH guidelines for the Care and Use of Laboratory Animals were followed along with the 2010/62/EU directive and the ARRIVE (Animal Research: Reporting of *In Vivo* Research: Reporting of *In Vivo* Experiments) guidelines. Syngeneic ID8-fluc cells were harvested using 0.05% Trypsin-EDTA and inoculated (5x10^6^ cells in 100µL DPBS) intraperitoneally in female C57BL/6 mice (six- to eight-week-old, obtained from Envigo (Horst, The Netherlands)), leading to the development of a stage III-IV ovarian cancer model (25).

Seven days post inoculation, treatment was initiated. Mice were divided into four groups receiving either: sham-heat and vehicle (n=8), sham-heat and olaparib (n=8), TTFields and vehicle (n=7) or TTFields and olaparib (n=11). TTFields treatment (200 kHz) was administered continuously using the inovivo^TM^ system (Novocure, Israel) by applying arrays to the shaven abdomen of the mice as previously described (30). Sham-heat used analogous non-therapeutic arrays. Olaparib (MedKoo Biosciences, USA) was dissolved in a vehicle of 10% DMSO, 50% PEG300 and 40% DPBS and administered at a concentration of 50 mg/kg/day through daily oral gavage. Overall, therapeutic interventions lasted for four weeks, given in four cycles of five consecutive treatment days, followed by two days without treatment.

### 2.12 Bioluminescent imaging

Tumor load was observed through bioluminescent imaging analysis before (day 5) and after (day 35) treatment administration. All mice were anaesthetized using isoflurane gas (2 L/min) and received 126 mg/kg of D-luciferin through subcutaneous injection. Ten minutes post injection, photon flux was measured using the IVIS-spectrum preclinical *In Vivo* Imaging System (Perkin-Elmer, USA). Normalized photon flux was calculated by subtracting the photon flux before treatment from the paired photon flux after treatment per mouse.

### 2.13 Overall survival

Mice were followed up and weighed daily once ascites development started as indicated by the appearance of abdominal distention. Mice were sacrificed when their body weight reached ≥32 grams as a surrogate endpoint for survival.

### 2.14 Statistical analysis

*In vitro* experiments were repeated at least three times, and data are presented as mean ± standard error of the mean (SEM), and analyzed with ANOVA or student’s t-test as appropriate. To determine the *in vivo* sample size, a statistical power analysis was performed to reach a power of at least 0.80. Photon-flux measured through BLI was summarized with means and standard deviations and visualized using bar charts. These results were analyzed using one-way ANOVA with Tukey’s multiple comparisons test. Kaplan-Meier curves were compared using the log-rank test. Multiple comparison adjustment was performed using the Benjamini-Hochberg procedure. Statistical analyses were performed using GraphPad Prism 10.1 software (La Jolla) and differences considered significant at (adjusted) p-values of: *p < 0.05, **p < 0.01, and ***p < 0.001.

## 3 Results

### 3.1 TTFields enhance the efficacy of carboplatin, additively in HRD cells and with a tendency to synergy in HRP and platinum-resistant cells

We examined carboplatin dose response curves, based on cell count measurements, in three different human ovarian cancer cell lines: A2780 (HRP cells), OVCAR-3 (HRD cells), and A2780cis (platinum-resistant cells, commercially available generated by repeated exposures of the A2780 cell line to cisplatin). The OVCAR-3 cells demonstrated highest sensitivity to carboplatin, while the A2780cis cells demonstrated highest resistance, as would be expected by the HRD phenotype of the former and the acquired resistance of the latter (**Supplementary Figure 1**).

**Figure 1:**
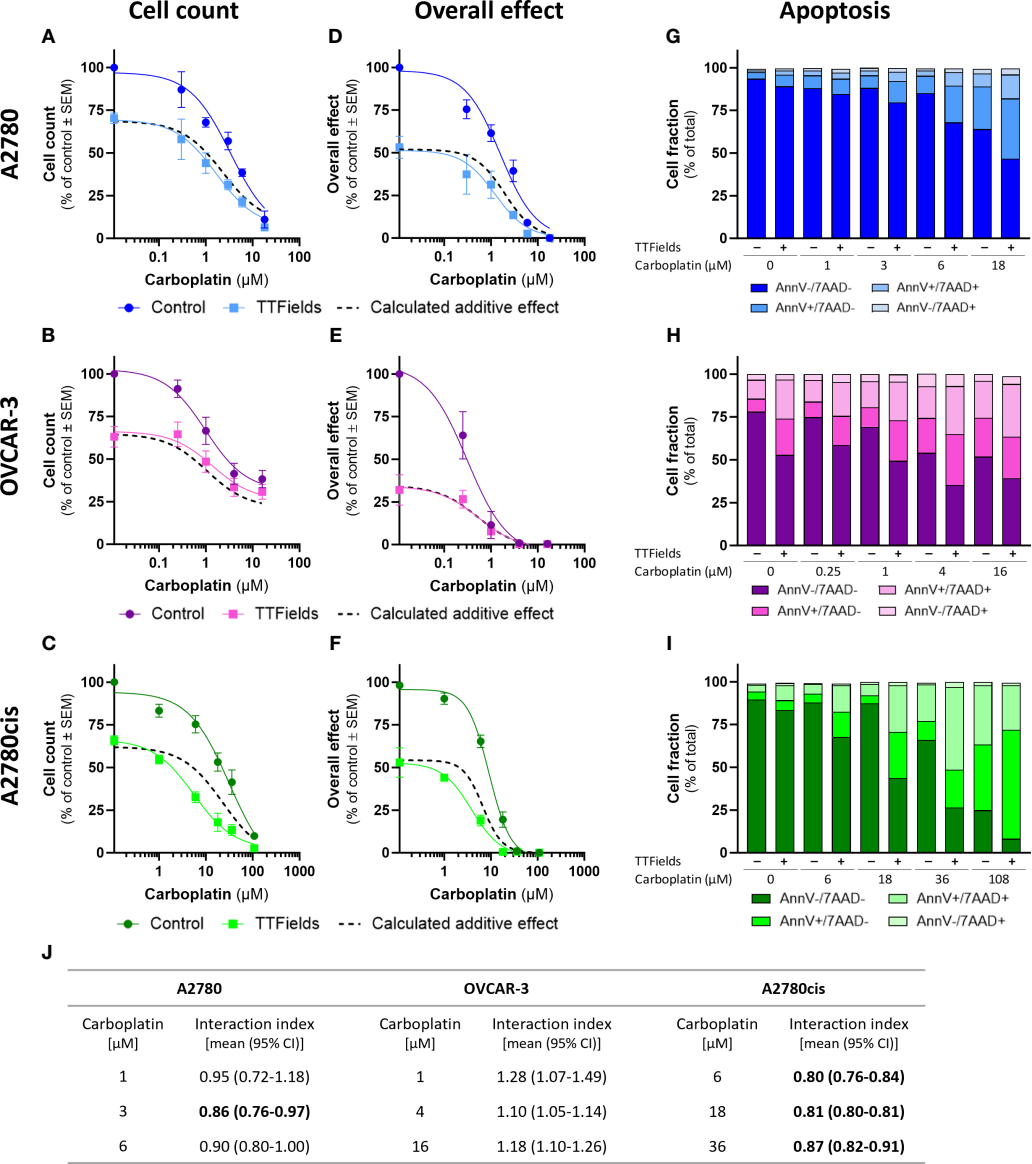
TTFields enhance the efficacy of carboplatin, additively in HRD cells and with a tendency to synergy in HRP and platinum-resistant cells. A2780 **(A, D, G)**, OVCAR-3 **(B, E, H)**, and A2780cis **(C, F, I)** human ovarian cancer cells (HRP, HRD, and platinum-resistant cells, respectively) were treated for 72 hr with various concentrations of carboplatin, alone or together with TTFields (200 kHz, 1 V/cm RMS), followed by examination of cell count **(A–C)**, overall effect **(D–F)**, and apoptosis **(G–I)**. Values are mean ± SEM. p<0.0001 for cell count and overall effect in all cell lines; Two-way ANOVA. Dashed lines represent the calculated additive effect, based on cell count and overall effect. For apoptosis: AnnV- 7AAD-, live cells; AnnV+ 7AAD-, cells at early apoptosis; AnnV+ 7AAD+, cells at late apoptosis. The interaction index (I_i_) for TTFields with various carboplatin concentrations was calculated by the Bliss independence method, and synergy denoted when 95% CI of I_i_ was lower than 1 (highlighted in bold) **(J)**.

TTFields significantly amplified (i.e. lower cell count) the effect induced by carboplatin alone in all examined cell lines (**Figures 1A–C**; p<0.0001 for all cell lines). Similarly, the overall effect (cell count × colony formation) induced by carboplatin was significantly elevated after addition of TTFields (**Figures 1D–F**; p<0.0001 for all cell lines). Apoptosis analysis demonstrated increases in the fraction of apoptotic cells when TTFields were applied with carboplatin, suggesting a cytotoxic effect (**Figures 1G–I**).

While the concomitant application of TTFields with carboplatin, led to enhanced treatment efficacy relative to each treatment alone, we further sought to elucidate the nature of interaction between the two modalities. We calculated the expected dose curve for an additive effect (**Figures 1A–F**, dashed lines) and the interaction index (I_i_, **Figure 1J**). An antagonistic interaction was demonstrated in OVCAR-3 cells, as the actual measured curves were above the calculated additive curves for co-treatment and the 95% confidence intervals (CI) for I_i_ were larger than 1. On the other hand, for A2780 and A2780cis cells synergy was determined, as the actual measured curves were below the calculated additive curves and the 95% CI for I_i_ were smaller than 1. The lower I_i_ determined for A2780cis relative to A2780 cells suggested higher levels of TTFields plus carboplatin synergy in A2780cis cells.

### 3.2 TTFields enhance the efficacy of PARPi, additively in HRD cells, with a tendency to synergy in platinum-resistant cells, and with high synergy in HRP cells

We next measured cell count dose response curves of olaparib and niraparib in the three different human ovarian cancer cell lines. As per the case with carboplatin, OVCAR-3 demonstrated highest sensitivity to PARPi while A2780cis demonstrated highest resistance (**Supplementary Figure 2**). This trait of A2780cis cells suggests that their acquired platinum resistance was also conferring some resistance to PARPi.

**Figure 2:**
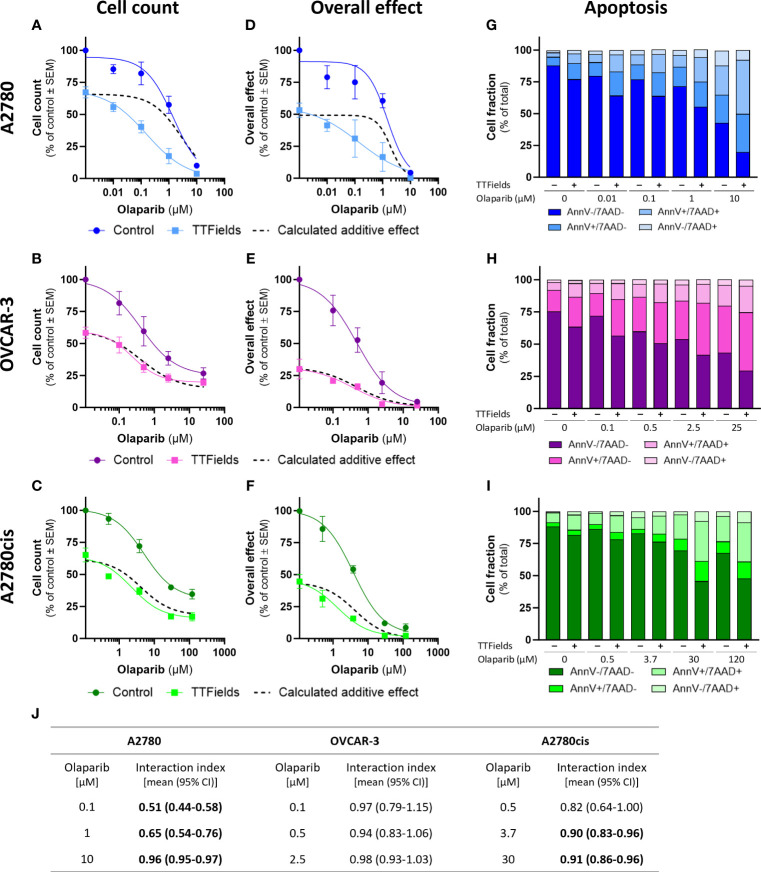
TTFields enhance the efficacy of olaparib, additively in HRD cells, with a tendency to synergy in platinum-resistant cells, and with high synergy in HRP cells. A2780 **(A, D, G)**, OVCAR-3 **(B, E, H)**, and A2780cis **(C, F, I)** human ovarian cancer cells (HRP, HRD, and platinum-resistant cells, respectively) were treated for 72 hr with various concentrations of olaparib, alone or together with TTFields (200 kHz, 1 V/cm RMS), followed by examination of cell count **(A–C)**, overall effect **(D–F)**, and apoptosis **(G–I)**. Values are mean ± SEM. p<0.0001 for cell count and overall effect in all cell lines; Two-way ANOVA. Dashed lines represent the calculated additive effect, based on cell count and overall effect. For apoptosis: AnnV- 7AAD-, live cells; AnnV+ 7AAD-, cells at early apoptosis; AnnV+ 7AAD+, cells at late apoptosis. The interaction index (I_i_) for TTFields with various olaparib concentrations was calculated by the Bliss independence method, and synergy denoted when 95% CI of I_i_ was lower than 1 (highlighted in bold) **(J)**.

**Figure 3:**
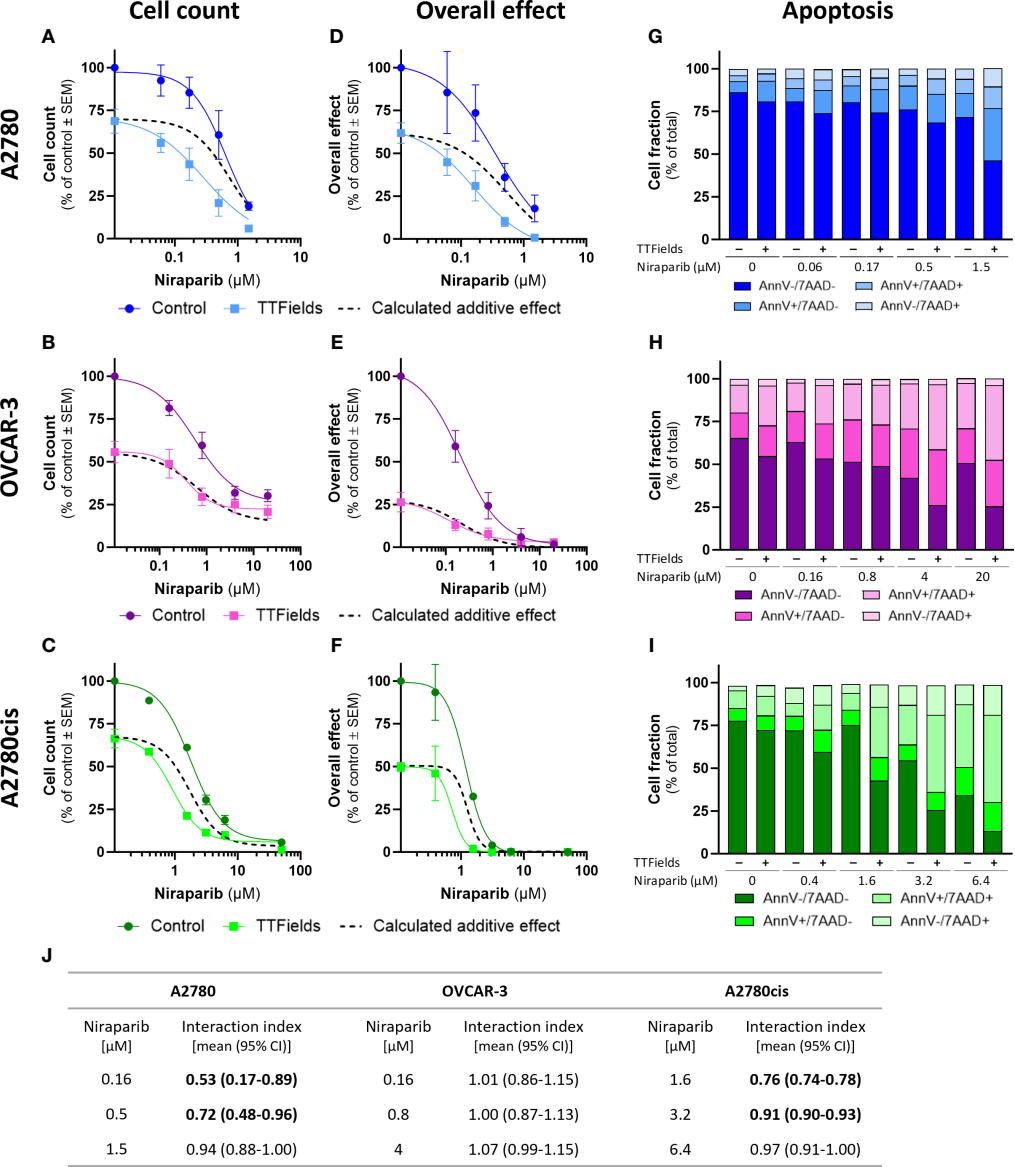
TTFields enhance the efficacy of niraparib, additively in HRD cells, with a tendency to synergy in platinum-resistant cells, and with high synergy in HRP cells. A2780 **(A, D, G)**, OVCAR-3 **(B, E, H)**, and A2780cis **(C, F, I)** human ovarian cancer cells (HRP, HRD, and platinum-resistant cells, respectively) were treated for 72 hr with various concentrations of niraparib, alone or together with TTFields (200 kHz, 1 V/cm RMS), followed by examination of cell count **(A–C)**, overall effect **(D–F)**, and apoptosis **(G–I)**. Values are mean ± SEM. p<0.0001 for cell count and overall effect in all cell lines; Two-way ANOVA. Dashed lines represent the calculated additive effect, based on cell count and overall effect. For apoptosis: AnnV- 7AAD-, live cells; AnnV+ 7AAD-, cells at early apoptosis; AnnV+ 7AAD+, cells at late apoptosis. The interaction index (I_i_) for TTFields with various niraparib concentrations was calculated by the Bliss independence method, and synergy denoted when 95% CI of I_i_ was lower than 1 (highlighted in bold) **(J)**.

TTFields significantly augmented the effect induced by olaparib and niraparib alone in all examined cell lines, as seen based on cell count (**Figures 2A–C**, **3A–C**, respectively; p<0.0001 for all cell lines with both drugs) and on the overall effect (**Figures 2D–F**, **3D–F**, respectively). Apoptosis analysis demonstrated elevation in the apoptotic cell fraction when TTFields were co-applied with olaparib or niraparib, suggesting a cytotoxic effect (**Figures 2G–I**, **3G–I**, respectively p<0.0001 for all cell lines with both drugs).

Additivity was determined for TTFields with PARPi in OVCAR-3 cells, as the actual measured curves overlapped the calculated additive curves for the co-treatment (and 95% CI for I_i_ spanned 1) (**Figures 2A–F**, **3A–F**, dashed lines; **Figures 2J**, **3J**). Different levels of synergy were determined in A2780 and A2780cis cells, with the lower I_i_ determined for the former indicating higher levels of TTFields plus PARPi synergy in A2780 cells. Interestingly, the levels of synergy for TTFields plus PARPi in the A2780 cells was higher than that demonstrated with carboplatin in either A2780 and A2780cis cells.

### 3.3 TTFields increase DNA damage induced by carboplatin and PARPi, downregulate the FA-BRCA pathway, and support drug-facilitated G2/M cell cycle arrest in response to the induced DNA damage

**Figure 4:**
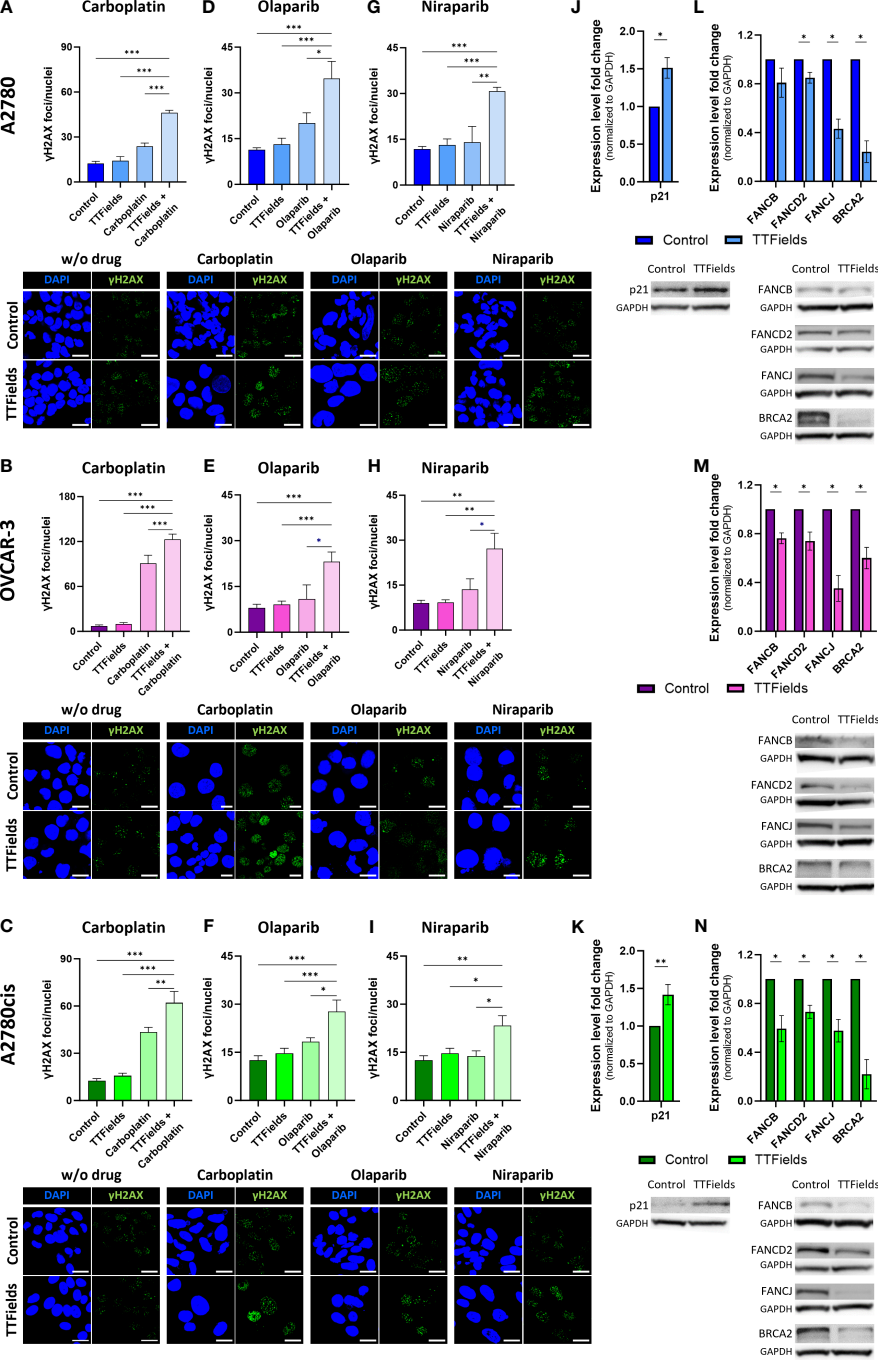
TTFields increase DNA damage induced by carboplatin and PARPi, elevate levels of p21, and downregulate the FA-BRCA pathway. A2780 **(A, D, G)**, OVCAR-3 **(B, E, H)**, and A2780cis **(C, F, I)** human ovarian cancer cells (HRP, HRD, and platinum-resistant cells, respectively) were treated for 72 hr with carboplatin **(A–C)**, olaparib **(D–F)**, or niraparib **(G–I)**, alone or together with TTFields (200 kHz, 1 V/cm RMS), followed by immunofluorescent detection of ɣH2AX foci formation. Drug concentrations: A2780 – 6 µM carboplatin, 1 µM olaparib, and 0.5 µM niraparib; OVCAR-3 – 16 µM carboplatin, 0.5 µM olaparib, and 0.8 µM niraparib; A2780cis – 36 µM carboplatin, 10 µM olaparib, and 1.5 µM niraparib. Representative images show staining with anti ɣH2AX antibody (green) and DAPI for nuclear visualization (blue) at x40 magnification; Scale bar, 20 µm. Values are mean ± SEM. *p < 0.05, **p < 0.01, and ***p < 0.001 relative to TTFields plus drug; One-way ANOVA, followed by Dunnett’s *post hoc* analysis. A2780 **(J)** and **(L)**, OVCAR-3 **(M)**, and A2780cis **(K)** and **(N)** human ovarian cancer cells were treated for 72 hr with TTFields (200 kHz, 1 V/cm RMS), followed by immunoblotting of cell lysates for expression of p21 **(J, K)**, FANCB, FANCD2, FANCJ, and BRCA2 **(L–N)**. Values are mean ± SEM. *p < 0.05, **p < 0.01, and ***p < 0.001 relative to control; Student’s T-tests.

We next examined accumulation of DNA damage in treated cells, by fluorescence microscopy detection of γH2AX foci in cell nuclei (**Figures 4A–I**). For these experiments, per each cell line, drugs were used at concentrations that induce 70 to 80 percent reduction in cell count when co-applied with TTFields. Under the selected conditions olaparib and niraparib alone, induced only a mild elevation in the levels of γH2AX in all cell lines. Carboplatin facilitated a more pronounced effect that was especially dramatic in the OVCAR-3 cells. Application of TTFields alone to the cells induced low or no effect on the level of γH2AX foci formation relative to control. However, co-application of TTFields together with either of the three drugs elevated the foci levels significantly relative to control and to TTFields or drug monotherapy.

To further understand how TTFields were involved in elevating DNA damage, we examined possible changes in expression levels of the cyclin-dependent kinase inhibitor p21 (**Figures 4J, K**), a key mediator of DNA damage-induced cell cycle arrest (31, 32), and of proteins from the FA-BRCA pathway for DNA damage repair (**Figures 4L–N**). TTFields application to the various cell lines elevated expression levels of p21 in A2780 and A2780cis cells (we could not detect p21 in the OVCAR-3 cells, as previously reported (33)), and decreased the expression of FANCB, FANCD2, FANCJ and BRCA2 relative to control cells in all three cell lines.

**Figure 5:**
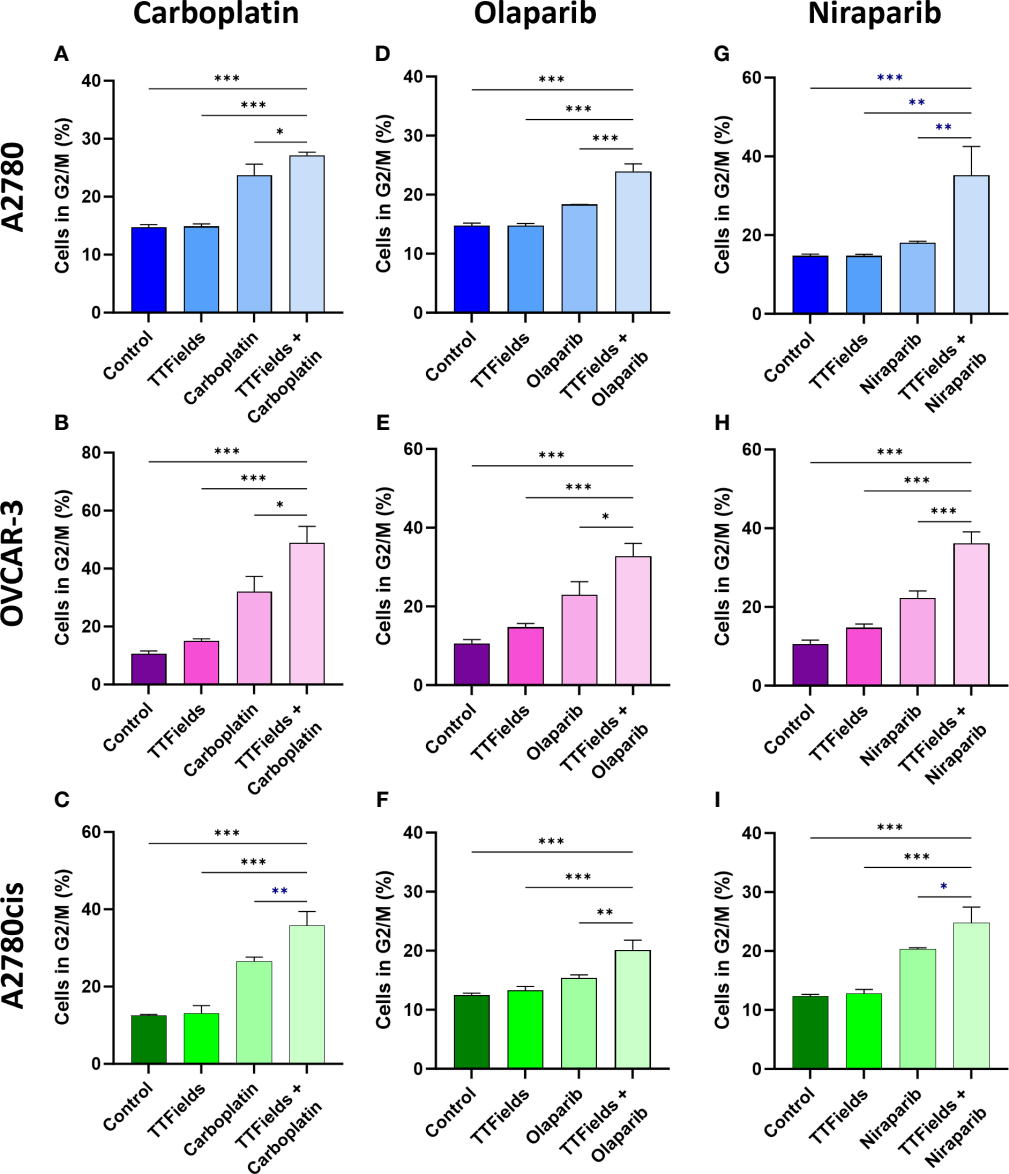
TTFields support drug-felicitated G2/M cell cycle arrest in response to the induced DNA damage. A2780 **(A, D, G)**, OVCAR-3 **(B, E, H)**, and A2780cis **(C, F, I)** human ovarian cancer cells (HRP, HRD, and platinum-resistant cells, respectively) were treated for 72 hr with carboplatin **(A–C)**, olaparib **(D–F)**, or niraparib **(G–I)**, alone or together with TTFields (200 kHz, 1 V/cm RMS), followed by staining with 7-AAD to determine the percentage of cells in G2/M phase. Drug concentrations: A2780 – 6 µM carboplatin, 1 µM olaparib, and 0.5 µM niraparib; OVCAR-3 – 16 µM carboplatin, 0.5 µM olaparib, and 0.8 µM niraparib; A2780cis – 36 µM carboplatin, 10 µM olaparib, and 1.5 µM niraparib. Values are mean ± SEM. *p < 0.05, **p < 0.01, and ***p < 0.001 relative to TTFields plus drug; One-way ANOVA, followed by Dunnett’s *post hoc* analysis.

We next tested whether the various treatments and their consequent DNA damage formation and p21 elevation could induce cell cycle arrest (**Figures 5A–I**; **Supplementary Figure 3**). Carboplatin, olaparib, and niraparib alone all induced G2/M arrest, while co-application of TTFields with each of the drugs significantly elevated the fraction of cells in G2/M.

### 3.4 TTFields co-treatment with olaparib inhibits tumor growth and prolongs survival in ovarian cancer bearing mice

**Figure 6:**
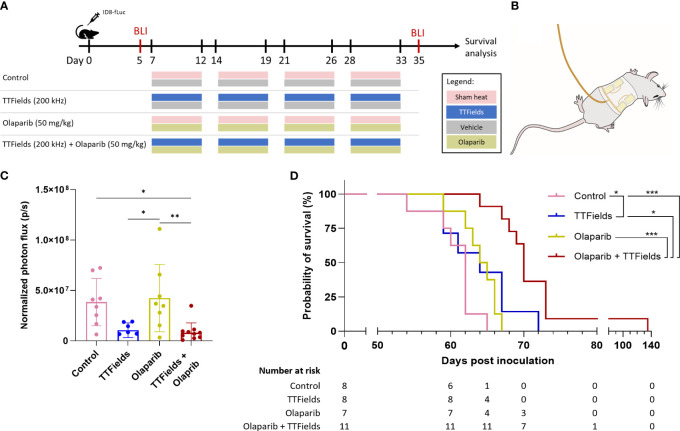
TTFields co-treatment with olaparib inhibits tumor growth and prolongs survival in ovarian cancer bearing mice. **(A)** *In vivo* treatment schedule. Mice were inoculated with ID8-fLuc (HRP) ovarian cancer cells, and treated with sham-heat and vehicle, sham-heat and olaparib (50 mg/kg/day), TTFields (200 kHz) and vehicle, or TTFields and olaparib. Treatments were administered five days a week for a period of four weeks starting on day 7 post inoculation. Bioluminescent imaging (BLI) was performed before start of treatment on day 5 and after treatment stop on day 35 post inoculation. **(B)** Illustration of TTFields/sham arrays applied to the mouse torso. The illustration depicted the dorsal electrodes. Ventral electrodes are positioned opposingly. **(C)** Normalized photon flux of paired mice for BLI before and after treatment. *adjusted p-value <0.05, **adjusted p-value <0.01, ****adjusted p-value<0.0001; one-way ANOVA with Tukey’s multiple comparisons test. **(D)** Kaplan-Meier curve showing the overall survival of the treated mice and number at risk. *adjusted p-value <0.05, ***adjusted p-value: 0.001; log-rank test, adjustments with the Benjamini-Hochberg procedure.

We measured the effect of TTFields application together with olaparib in mice bearing orthotopic ID8-fLuc (HRP cells) ovarian cancer tumors. Experimental timeline and a schematic illustration of the TTFields/sham arrays attached to the mouse torso are depicted in **Figures 6A, B**, respectively. The photon flux measured before treatment showed 100% tumor engraftment, and no significant difference in tumor volume between treatment groups at the start timepoint (**Supplementary Figure 4**). After the 4 weeks treatment period, significantly smaller tumor volumes were seen for mice treated with TTFields plus olaparib, which were lower by about 80% compared to controls (p=0.0183) and to olaparib only treated mice (p=0.0066) (**Figure 6C**). Additionally, about 70% reduction in tumor growth was observed in mice treated with TTFields alone compared to olaparib monotherapy (p=0.0288) and to control (p=0.0658).

The Kaplan-Meier curve showed median overall survival of 62 days for control, 64 days for TTFields alone, 64.5 days for olaparib alone, and 70 days for TTFields plus olaparib (**Figure 6D**). Survival was significantly prolonged in the mice co-treated with TTFields plus olaparib compared to control mice (p=0.0003), olaparib monotherapy (p=0.0003), and TTFields monotherapy (p=0.0130). Notably, survival benefit was also observed in olaparib only treated mice compared to control mice (p=0.0401).

## 4 Discussion

The efficacy of TTFields for treatment of ovarian cancer has previously been demonstrated in preclinical models and in the INNOVATE study (34, 35). In those studies, co-application of TTFields with paclitaxel was tested, as to take advantage of the antimitotic effects manifested by both treatment modalities (36). With the recently identified effect of TTFields on DNA damage and repair (14–17, 37), we sought to examine the effect of adding TTFields to current treatments used for ovarian cancer which target such pathways. Specifically, the effects of TTFields concomitant with carboplatin and PARPi were investigated in HRP, HRD, and platinum-resistant cells.

While TTFields enhanced the efficacy of all the drugs tested in this study in all the examined cell lines, the nature of the interaction was found to be dependent on the drug’s underlying mechanism of action, as well as on the genetic background of the cells. In HRD cells (OVCAR-3), carboplatin interacted with TTFields antagonistically, while PARPi interacted with TTFields additively. In HRP cells (A2780) and in platinum-resistant cells (A2780cis), however, all drugs interacted with TTFields synergistically. Interestingly, TTFields-carboplatin synergy was higher in A2780cis relative to A2780 cells, whereas synergy for TTFields with either of the two PARPi (olaparib and niraparib) was higher in A2780 relative to A2780cis cells. Additionally, TTFields with PARPi reached higher levels of synergy relative to TTFields with carboplatin. Overall, these results suggest a potential benefit for concurrent application of TTFields with carboplatin or PARPi in ovarian cancer.

Treatment of the cells with TTFields plus carboplatin or PARPi demonstrated elevated levels of DNA damage, increased expression of p21, a CDK inhibitor involved in induction of cell cycle arrest in response to DNA damage, and elevated G2/M cell cycle arrest. To shed light on this outcome, we tested the effects of TTFields on the expression of proteins from the FA-BRCA pathway, previously shown to be downregulated by TTFields in other tumor types (14–17). Indeed, TTFields application resulted in decreased expression of FANCB, a protein involved in the FA core complex, FANCD2 from the FANCI-FANCD2 (ID) complex, and of the two pivotal downstream proteins FANCJ and BRCA2, suggesting that the FA-BRCA pathway was severely impaired. While this effect was seen in all the examined cell lines, the manifestation of the effect was dependent on the co-applied drug and the genetic background of the cells, as detailed below.

The differences in the interactions between TTFields and the drugs seen for the different cell lines could be explained based on the HRP/HRD status of the cells. In the HRP cells (A2780), applying TTFields induced a state of BRCAness, hence creating synthetic lethality with PARPi, resulting in a highly synergistic effect. However, in the HRD cells (OVCAR-3), synthetic lethality with PARPi stemmed from the genetic background of the cells, and so the added effect of TTFields on FA-BRCA protein downregulation was transparent. Still, an additive effect for TTFields with PARPi was demonstrated in the HRD cells (OVCAR-3), which may relate to effects of TTFields on cancer cells unrelated to DNA damage repair mechanisms, such as the antimitotic effect (11–13). Additionally, TTFields have been shown in GBM cells to increase cell membrane permeability (38), an effect that was suggested to increase cellular drug uptake, and warrant further examination.

The resolution of DNA damage induced by carboplatin involves multiple factors from different repair pathways, mainly the FA-BRCA pathway and nucleotide excision repair (NER). Synergy between TTFields and carboplatin in the HRP cells (A2780) may therefore be related to this treatment regimen inducing damage while simultaneously blocking one of the pathways needed for its repair. Such cellular conditional vulnerability, with synergy between TTFields and cisplatin, has previously been demonstrated in pleural mesothelioma (17). The conditional vulnerability instated by the TTFields-induced BRCAness state is however not limited to platinum-based agents, and can also be exploited for concomitant use with other cancer treatment modalities that induce DNA damage. The benefit of applying TTFields with radiation was already demonstrated preclinically (14, 15, 37, 39); and the potential of this treatment option in patients with newly-diagnosed GBM is currently under clinical investigation (TRIDENT, NCT04471844) (40, 41).

Platinum resistance is a strong predictive marker for PARPi resistance, indicating inter-related mechanisms (2, 3, 42). Indeed, the platinum-resistant cell line used in this study (A2780cis) also demonstrated PARPi resistance relative to its parental cell line. Drug resistance mechanisms are complex, encompassing changes in cellular availability of the drugs and alternation in DNA damage response (2, 3, 42). Changes in the capacity to repair DNA damage were previously demonstrated for A2780 cells resistant to cisplatin compared to their parental cells (43, 44). Therefore, modulation of DNA damage repair can potentially account for the differences observed in the interaction of TTFields and the drugs between the resistant and the parental A2780 cell lines used in this study. The observation that TTFields application to resistant cells could sensitize them to platinum-based chemotherapy and PARPi is encouraging and may have clinical translation. However, to fully understand the type of TTFields-drug interactions in the resistant cells, in depth genetic characterization of these cells is needed.

The *in vivo* experiments conducted in this research focused on application of TTFields plus olaparib to ovarian cancer HRP cells, the case which showed highest benefit in the *in vitro* setting. Results showed that TTFields were effective in reducing ID8 tumor growth relative to control, while olaparib was not, in accordance with previous reports (45, 46). When TTFields and olaparib were applied together, significant reduction in tumor growth was observed relative to treatment with olaparib alone and to control. Surprisingly, while TTFields plus olaparib showed similar reduction in tumor growth as TTFields alone, a significant improvement relative to the monotherapies was observed when TTFields were applied together with olaparib in regard to overall survival, confirming the beneficial effect of this treatment regimen. This outcome can be explained by effects that come into play in longer timeframes, such as alternations in the systemic anti-tumor immune response.

PARPi have been shown to have immunostimulating mechanisms, including activation of the cGAS/STING pathway in cancer cells (47, 48). TTFields were also shown to induce immunogenic cell death and cGAS/STING activation in preclinical models and in the clinic (30, 49, 50). Future studies will hence focus on the immunological aspects of applying TTFields together with olaparib in ovarian cancer, and on the potential use of TTFields together with both PARP and immune checkpoint inhibitors.

In conclusion, platinum-based chemotherapy and PARP inhibition are effective mainly in patients with HRD tumors, while patients with HRP tumors show treatment resistance (51). TTFields induce an HRD-like phenotype, manifesting synergy with the aforementioned drugs, showing potential for ovarian cancer treatment throughout the adjuvant and maintenance stages, in both HRP and HRD ovarian cancer cells, as well as in cells with treatment resistance. As a-priori and *de novo* drug resistance are a major limitation in ovarian cancer treatment, TTFields-induced sensitization of HRP cells and cells with acquired drug resistance can thus potentially help mitigate the problem.

## Data availability statement

The original contributions presented in the study are included in the article/**Supplementary Material**. Further inquiries can be directed to the corresponding author.

## Ethics statement

Ethical approval was not required for the studies on humans in accordance with the local legislation and institutional requirements because only commercially available established cell lines were used. The animal study was approved by KU Leuven ethical committee (P082/2021). The study was conducted in accordance with the local legislation and institutional requirements.

## Author contributions

YB: Conceptualization, Investigation, Validation, Visualization, Writing – review & editing, Formal analysis. HE: Conceptualization, Formal analysis, Investigation, Project administration, Validation, Visualization, Writing – review & editing. KB: Conceptualization, Formal analysis, Investigation, Validation, Visualization, Writing – review & editing. AM: Conceptualization, Formal analysis, Investigation, Project administration, Validation, Visualization, Writing – review & editing. RW: Conceptualization, Investigation, Writing – review & editing. BV: Investigation, Writing – review & editing. SV: Investigation, Writing – review & editing. RM: Investigation, Writing – review & editing, Formal analysis, Validation, Visualization. RF: Visualization, Investigation, Writing – review & editing, Formal analysis, Validation. HG: Formal analysis, Validation, Visualization, Investigation, Writing – review & editing. ED: Writing – review & editing, Project administration, Conceptualization. AH: Formal analysis, Validation, Visualization, Writing – original draft, Writing – review & editing. MG: Conceptualization, Supervision, Writing – review & editing. UW: Writing – review & editing. IV: Writing – review & editing. AC: Supervision, Writing – review & editing, Conceptualization. YP: Writing – review & editing.
