# Supplementary material for: Tumor Treating Fields (TTFields) induce homologous recombination deficiency in ovarian cancer cells, thus mitigating drug resistance
Source: Front Oncol. 2024 Jun 27;14:1402851. doi: 10.3389/fonc.2024.1402851 (PMC11238040; doi:10.3389/fonc.2024.1402851)

Figure S1. Carboplatin dose response curves in the three different cell lines. Cell count of A2780, OVCAR-3, and A2780cis human ovarian cancer cells (HRP, HRD, and platinum-resistant cells, respectively) following 72 h treatment with various carboplatin doses. Values are mean ± SEM.

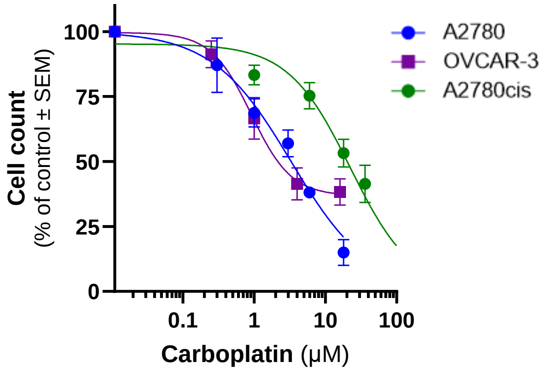

Figure S2. Olaparib and niraparib dose response curves in the three different cell lines. Cell count of A2780, OVCAR-3, and A2780cis human ovarian cancer cells (HRP, HRD, and platinum-resistant cells, respectively) following 72 h treatment with various olaparib **(A)** or niraparib **(B)** doses. Values are mean ± SEM.

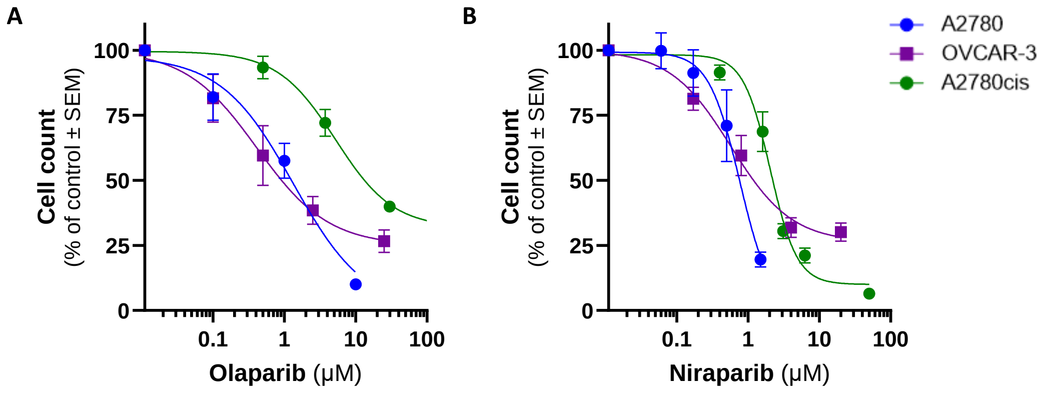

Figure S3. Complementary data for the cell cycle analysis. Percentage of cells in G0/G1 and S phases for the data shown in Figure 5.

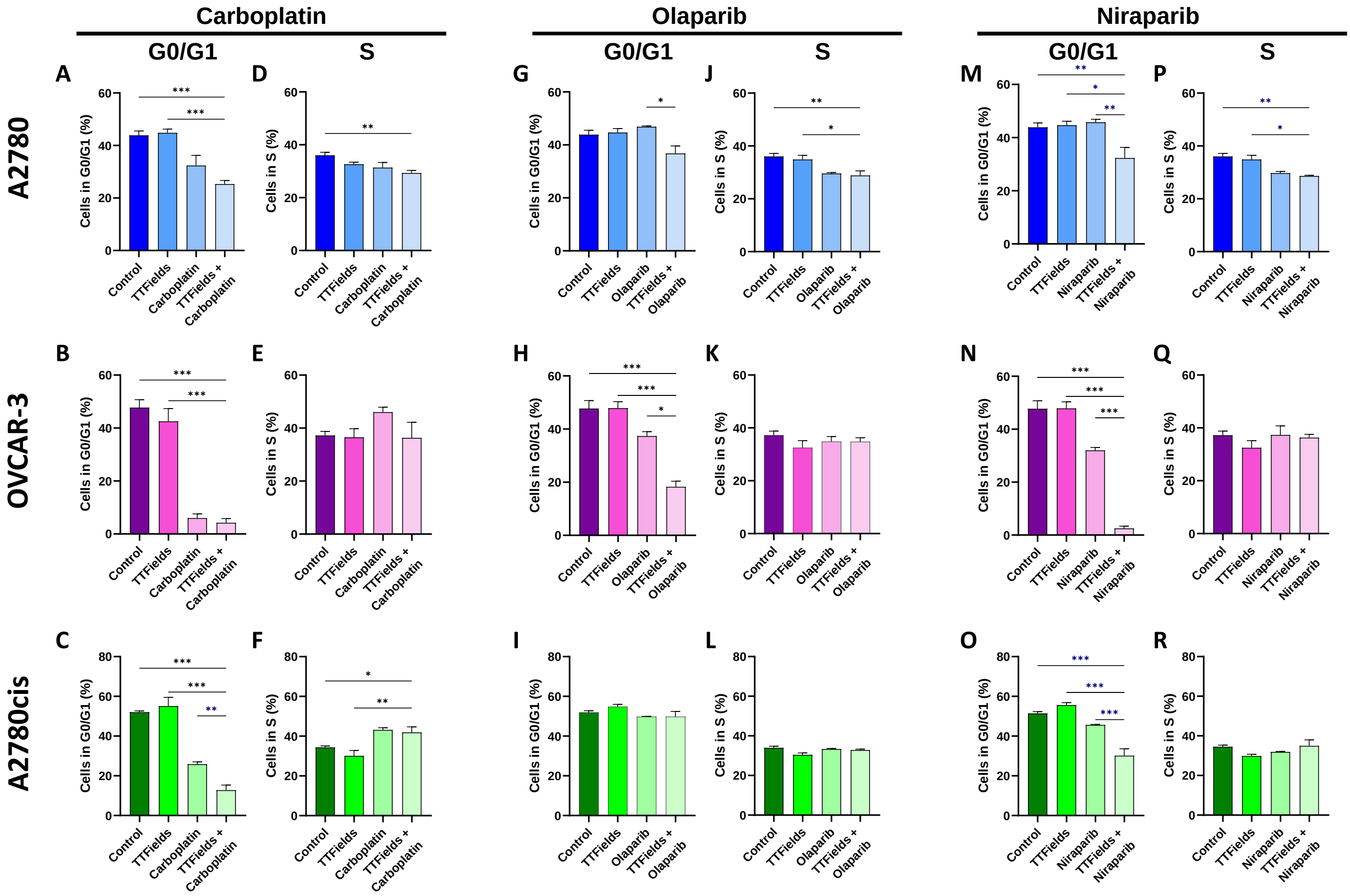

Figure S4. Complementary data for in vivo study. Total photon flux of the bioluminescent imaging analysis before and after treatment.

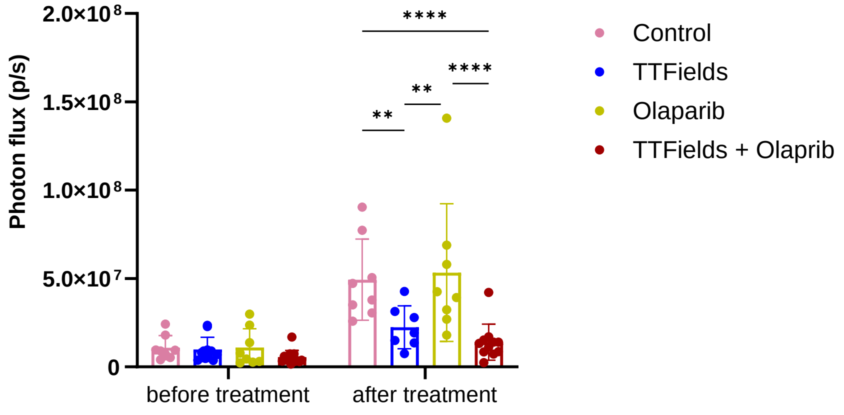

Supplement: Supplementary file 1 [file DataSheet_1.pdf]
